# Second language learning in older adults modulates Stroop task performance and brain activation

**DOI:** 10.3389/fnagi.2024.1398015

**Published:** 2024-08-07

**Authors:** Douglas H. Schultz, Alison Gansemer, Kiley Allgood, Mariah Gentz, Lauren Secilmis, Zoha Deldar, Cary R. Savage, Ladan Ghazi Saidi

**Affiliations:** ^1^Department of Psychology, University of Nebraska-Lincoln, Lincoln, NE, United States; ^2^Center for Brain, Biology and Behavior, University of Nebraska-Lincoln, Lincoln, NE, United States; ^3^Department of Communication Disorders, College of Education, University of Nebraska at Kearney, Kearney, NE, United States; ^4^Department of Psychology, McGill University, Montreal, QC, Canada

**Keywords:** language learning, cognitive effects, Stroop task, older adults, aging, cognitive reserve, fMRI

## Abstract

**Introduction:**

Numerous studies have highlighted cognitive benefits in lifelong bilinguals during aging, manifesting as superior performance on cognitive tasks compared to monolingual counterparts. Yet, the cognitive impacts of acquiring a new language in older adulthood remain unexplored. In this study, we assessed both behavioral and fMRI responses during a Stroop task in older adults, pre- and post language-learning intervention.

**Methods:**

A group of 41 participants (age:60–80) from a predominantly monolingual environment underwent a four-month online language course, selecting a new language of their preference. This intervention mandated engagement for 90 minutes a day, five days a week. Daily tracking was employed to monitor progress and retention. All participants completed a color-word Stroop task inside the scanner before and after the language instruction period.

**Results:**

We found that performance on the Stroop task, as evidenced by accuracy and reaction time, improved following the language learning intervention. With the neuroimaging data, we observed significant differences in activity between congruent and incongruent trials in key regions in the prefrontal and parietal cortex. These results are consistent with previous reports using the Stroop paradigm. We also found that the amount of time participants spent with the language learning program was related to differential activity in these brain areas. Specifically, we found that people who spent more time with the language learning program showed a greater increase in differential activity between congruent and incongruent trials after the intervention relative to before.

**Discussion:**

Future research is needed to determine the optimal parameters for language learning as an effective cognitive intervention for aging populations. We propose that with sufficient engagement, language learning can enhance specific domains of cognition such as the executive functions. These results extend the understanding of cognitive reserve and its augmentation through targeted interventions, setting a foundation for future investigations.

## 1 Introduction

In recent years, the prevention of cognitive decline in normal or pathological aging has emerged as a paramount focus of many neuropsychological studies (Di Nuovo et al., 2020; Oosterhuis et al., 2023). This is particularly important given the increasing aging population (Kanasi et al., 2016; Osareme et al., 2024) and the prevalence of age-related cognitive disorders (Lopez and Kuller, 2019). The role of non-pharmaceutical interventions in mitigating the progression of cognitive decline is crucial. Unlike pharmacological treatments, which often target specific symptoms or stages of cognitive diseases, non-pharmaceutical interventions offer a holistic approach with the potential for broader applicability and fewer side effects (Dyer et al., 2018). Non-pharmaceutical interventions which encompass a variety of options from lifestyle modifications to cognitive training (Šneidere et al., 2024); and dietary adjustments are considered proactive strategies to preserve cognitive function (Klimova et al., 2017) and strengthen cognitive reserve (Bott et al., 2019).

Cognitive reserve, a fundamental concept in healthy aging, refers to the brain’s resilience to neuropathological damage (Scarmeas and Stern, 2003; Cammisuli et al., 2022; Savarimuthu and Ponniah, 2024). Emerging evidence suggests that higher cognitive reserve is associated with greater ability to compensate for age-related brain changes and pathology, thereby mitigating the manifestation of clinical symptoms (Tucker and Stern, 2011). This resilience is believed to stem from a variety of life-long experiences, including education, occupational complexity, and engagement in cognitively stimulating activities such as music, games, reading and so forth (Liberati et al., 2012; Savarimuthu and Ponniah, 2024).

Among the factors contributing to cognitive reserve, bilingualism has emerged as a significant area of interest in the past decade (Bialystok, 2021). There is behavioral and neuroimaging evidence that lifelong bilingualism enhances cognitive reserve (Craik et al., 2010; Schweizer et al., 2012). Bilingual individuals engage in constant cognitive exercises, such as switching between languages and inhibiting one language while using another (Green and Abutalebi, 2013). This cognitive exercise over a lifetime is believed to strengthen neural networks and enhance cognitive flexibility (Barbu et al., 2018). Further, there is evidence that the cognitive demand associated with managing two languages contributes to the development of higher cognitive reserve (Bialystok et al., 2007; Gallo and Abutalebi, 2024) and therefore delaying the onset of age-related cognitive decline and neurodegenerative diseases (Bialystok et al., 2016, 2024).

Many researchers agree on the underlying reasons why bilingualism might contribute to enhanced cognitive reserve. Engaging in bilingualism requires the navigation of complex linguistic structures, managing two linguistic systems simultaneously, requiring inhibitory control and therefore continuously engaging executive control functions, such as task switching and inhibition (Kroll and Bialystok, 2013; Gallo and Abutalebi, 2024). These cognitive demands are hypothesized to lead to structural and functional brain changes, including in areas involved in executive control and language processing (García-Pentón et al., 2014). Additionally, the constant management of dual language systems is believed to enhance neural plasticity and efficiency. This may provide a neurological buffer against age-related cognitive decline (Del Maschio et al., 2018).

Despite the body of evidence for cognitive advantages of lifelong bilingualism in aging, research on the cognitive effects of second language (L2) acquisition in older adults is still in its infancy. In a recent systematic review on the role of bilingualism in executive functions in healthy older adults, out of 24 studies, nine provided full support for the bilingual advantage, while four indicated a bilingual disadvantage. The remaining studies neither fully supported nor refuted the bilingual advantage, presenting mixed results across various domains. However, when analyzing specific domains of executive functions, bilingualism is consistently associated with an advantage in inhibition, particularly evidenced by performance in the Stroop test and the Simon task (Degirmenci et al., 2022).

A few studies have reported that L2 learning in older adults can lead to improvements in global cognition (Antoniou et al., 2013) and specific cognitive tasks such as memory, attention, and executive functioning (Meltzer et al., 2023), potentially offering a protective mechanism against cognitive decline. This enhancement is often attributed to the cognitive stimulation and neural plasticity induced by the complexities of acquiring a new language (Li et al., 2014), which in turn may boost cognitive reserves (Antoniou et al., 2013).

The cognitive demands of second language (L2) acquisition is well established by theoretical models such as the dynamic model (Abutalebi and Green, 2007; Korenar and Pliatsikas, 2023), and experimental research (Ghazi Saidi et al., 2013; Fukuta and Yamashita, 2015; Ghazi-Saidi and Ansaldo, 2017a,b; Li and Xu, 2023). Learning a new language entails extensive engagement of various cognitive systems, particularly those involved in memory, attention, and executive functioning (Ghazi Saidi et al., 2013; Ghazi-Saidi and Ansaldo, 2017a,b). This engagement is hypothesized to not only activate and strengthen these cognitive areas but also potentially induce neuroplastic changes within the brain.

The Stroop task is an excellent choice for measuring the cognitive effects of language learning for several reasons. First, the Stroop task is designed to assess executive function, particularly cognitive control and inhibition (Egner and Hirsch, 2005; Guarino et al., 2020). Learning a new language requires similar cognitive skills, such as controlling attention between different language systems and inhibiting one language system while using another (Kroll et al., 2008; Green and Abutalebi, 2013; Hirosh and Degani, 2018). The Stroop task is also known to activate several brain regions associated with language processing, attention, and executive function (Ye and Zhou, 2009; Heidlmayr et al., 2020). Therefore, learning a new language may improve performance on the Stroop task. Second, given that Stroop task involves processing words and colors, it inherently taps into language processing areas of the brain (Hertrich et al., 2021). Further, the Stroop test has been identified as a tool to measure cognitive health at aging (Gajewski et al., 2020). Stroop has been recommended as an efficient tool for early detection of cognitive impairment in individuals with subjective cognitive complaints (Rao et al., 2023). In addition, Stroop has been identified as a tool to distinguish individuals with Mild Alzheimer’s disease (AD) from non-AD individuals as well as mild versus severe AD (Lin and Lai, 2024). Cognitive aging is associated with compromised interference control (Gajewski et al., 2020; Rao et al., 2023; Lin and Lai, 2024). Thus, the Stroop task provides an objective measure to assess a specific cognitive domain at baseline and after the intervention. Any significant change in performance on the Stroop task from pre to post intervention can be attributed to cognitive changes potentially induced by the language learning process.

## 2 Methods

### 2.1 Design and procedure

In this study, we used a pre-post intervention design with functional magnetic resonance imaging (fMRI) to explore the neural effects of language learning in older adults. The study was initiated by conducting baseline fMRI scans for all participants to establish a neurological benchmark. Following this, the participants underwent an online language learning program. Participants were monitored daily for their performance and retention. After completion of the intervention, a second set of fMRI scans was conducted to identify any neural changes attributable to the intervention. This pre-post design allows for the assessment of neural adaptations in response to new language acquisition in older adults. The fMRI data were analyzed using standard neuroimaging techniques to observe changes in brain activity, with a particular focus on regions associated with language processing and cognitive control.

This study was approved by the UNL Institutional Review Board (IRB), Research Compliance Services, Office of Research & Economic Development, University of Nebraska-Lincoln. The University of Nebraska at Kearney IRB acknowledged and honored the site agreement to cede IRB review to the University of Nebraska-Lincoln IRB regarding this study, under the SMART IRB Master Common Reciprocal Institutional Review Board Authorization Agreement. Per this agreement the UNL IRB serves as the reviewing IRB and the UNK IRB as the relying IRB.

### 2.2 Participants

Our participant cohort consisted of 41 healthy, monolingual individuals, aged between 60 and 80 years (*M* = 66.63, SD = 4.7, min = 60; max = 77), all residing in a predominantly monolingual environment in Nebraska. All participants had at least 14 years of education (*M* = 17.5, SD = 2.94). All participants were white caucasian, mirroring the demographics of the rural Nebraskan population. All ethnic minorities were excluded based on proficiency in two or more languages.

### 2.3 Inclusion and exclusion criteria

Inclusion criteria consisted of being monolingual English-speaking adults, aged 60–80 years of any gender, race or ethnicity, with an electronic device and access to the internet, with normal or corrected vision and hearing, no memory or learning problems, no diagnosed depression or neurological disorders. In addition, we only included right-handed individuals to control for left-hemisphere dominance. All participants were fully vaccinated for COVID-19 given that data were collected shortly after COVID-19 restrictions were lifted to avoid putting older adults at additional risk of exposure to COVID-19. Participants who were claustrophobic or did not have the ability to report to the Center for Brain, Biology and Behavior in Lincoln, NE, or did not pass the MRI compatibility screener were excluded. All participants had computer skills to enroll in the intervention program and access to the internet and a device. All participants were screened using Montreal Cognitive Assessment (MoCA) and participants who did not score above the cut-off of 26 were excluded.

The Language Experience & Proficiency Questionnaire (LEAP-Q) (Marian et al., 2007; Kaushanskaya et al., 2020) was used to screen for second language knowledge, use and exposure. LEAP-Q is a self-report toolbox to collect data on language knowledge, use and exposure for all languages spoken by an individual. Scores range 0–10, and scores above 7 are taken as a measure of bilingual proficiency; a score of 0 indicates monolingualism. Only participants with a score of 0 or 1 (minimal knowledge and at vocabulary level) were included.

### 2.4 Intervention

Participants engaged in an online language learning program, Rosetta Stone^1^, in which they selected a language of their choice to learn. Rosetta Stone is a comprehensive computer-assisted language learning software developed by Rosetta Stone Ltd. This software employs an immersive method, inspired by the naturalistic way individuals learn their first language (Work, 2014; Zheng, 2024). The approach is characterized by the absence of translations or explicit grammar instructions, instead relying on visual, auditory, and textual cues in the target language to convey meaning and foster language comprehension and production. This methodology aligns with the communicative approach to language teaching, which emphasizes the importance of interaction and using the language for real-life communication purposes. This method mirrors the way a baby learns their mother tongue, emphasizing immersion and natural acquisition. As a result, the writing system is initially excluded from the learning process. For this reason, we did not exclude languages with different writing systems. We used the educational version of Rosetta Stone which provided us with the possibility of monitoring the adherence to the program with detailed information about the number of minutes engaged in the program, a detailed statistics about the activities in which the participant engaged, the number of times each activity was repeated and scores on tests at the end of each lesson and each level. Among the 26 different options available, participants selected Spanish, French, German, Italian, and Japanese, in order of popularity.

The cognitive effects of second language (L2) learning are not directly tied to the specific language being learned. Instead, L2 learning enhances broader cognitive functions, including memory, attention, problem-solving, and executive functions (Ghazi Saidi et al., 2013; Guo and Ma, 2023). These cognitive processes are fundamental to overall cognitive functioning and are not exclusive to the specific L2. Managing two linguistic systems improves executive control mechanisms, such as task switching, inhibitory control, and cognitive flexibility, which are general cognitive skills (Gallo and Abutalebi, 2024; Han et al., 2024). Consequently, the cognitive benefits of L2 learning, such as improved working memory and attentional control (Bialystok and Craik, 2022), are transferable to other cognitive domains, enhancing overall cognitive performance (Waldie et al., 2009; Antoniou, 2019; Gallo and Abutalebi, 2024; Han et al., 2024).

The intervention spanned a duration of 4 months. Participants were instructed to engage in language learning 5 days per week, at a dose of 90 min per day. The online platform provided flexibility for participants to learn at their own pace and in a familiar environment, potentially enhancing adherence to the program. To ensure a consistent and effective learning experience, we offered optional monthly zoom meetings to participants. These meetings provided them with an opportunity to meet other participants, ask questions and share their experiences. Adherence to the intervention protocol was closely monitored. As administrators, we had access to log-in and time and type of language learning activities. We collected data on daily activities, and test results. At the end of each lesson, there was a test. Participants could proceed with the lesson only if they passed the test with 80% accuracy or above. This data was then used to assess the fidelity of the intervention and its potential impact on the cognitive abilities of the participants.

### 2.5 Pre/post Assessment

The Pre/Post assessment included in the scanner was the Stroop task, that measures cognitive control and executive function (Kane and Engle, 2003; Bari and Robbins, 2013). The assessment task was conducted in English, the participants’ mother tongue. Testing in the participants’ dominant language is crucial for the accuracy of neuropsychological assessment results (e.g., Brown and Weisman de Mamani, 2017; Momková and Jurásová, 2017; Stålhammar et al., 2022).

### 2.6 Stroop task

Two primary hypotheses have been proposed to explain the cognitive effects of bilingualism. The Bilingual Inhibitory Control Advantage (BICA) hypothesis suggests that bilinguals exhibit better performance in tasks requiring conflict resolution, such as incongruent conditions in Stroop or flanker tasks. On the other hand, the Bilingual Executive Processing Advantage (BEPA) hypothesis proposes a broader cognitive benefit, indicated by faster response times in both congruent and incongruent trials of cognitive control tasks. Evidence exists supporting both hypotheses (Hilchey and Klein, 2011). This study is based on the BICA hypothesis, as the Stroop task is more extensively supported in the literature compared to other tasks (Waldie et al., 2009; Rodríguez-Pujadas et al., 2014; Costumero et al., 2015; Teubner-Rhodes et al., 2019).

The color-word Stroop task (Stroop, 1935; Scarpina and Tagini, 2017) is a well-established neuropsychological test designed to evaluate cognitive control and executive function (Kane and Engle, 2003; Bari and Robbins, 2013). During the Stroop task, participants are presented with words denoting colors that are printed in congruent or incongruent colors (e.g., the word “red” printed in blue). They were required to identify the color of the “ink,” not the word itself, which requires the inhibition of an automatic reading response. Specifically, the congruent trials included the names of colors that appeared in the same color that they read. The incongruent trials included the names of colors that appeared in a different color that they read. The participants were instructed to always pick the color of the word on the monitor (i.e., the “ink”), and ignore what the word “read.” The neutral condition consisted of the words “when,” “so” and “for” which were presented in different colors on the monitor. Participants were instructed to respond with a right index finger key press for when the color of the word was yellow, a right middle finger key press when the color of the word was red, and a right ring finger key press when the color of the word was green. The task was displayed to participants via a screen visible through a mirror mounted on the head coil.

The Stroop task paradigm was previously used in a number of studies (MacLeod, 1991; Kane and Engle, 2003). Our custom version of the Stroop task consisted of 108 trials, divided into three conditions with each condition consisting of 36 trials. Stimuli were presented with E-prime version 2.0 software (Psychology Software Tools, Pittsburgh, PA). Trials were presented in a pseudorandom order to control for sequence effects: (1) Neutral Condition: Departing from the traditional non-word letter strings, our task incorporated common English words such as “When,” “So,” and “Like.” These words, which do not inherently relate to colors, were presented in yellow, red, or green. This adaptation aimed to control the impact of familiarity and semantic content on the participants’ response times and accuracy. (2) Congruent Trials: For these trials, color words such as “Yellow,” “Red,” and “Green” were displayed in their respective colors. The semantic and visual congruence would expedite response times, leveraging the alignment between the word meaning and its visual presentation. (3) Incongruent Trials: These trials featured color words in contrasting colors, thereby inducing the Stroop effect. Each color word was repeated 12 times in each of the three incongruent colors, resulting in a total of 36 trials for each condition.

Each trial commenced with blank screen for 1,000 milliseconds (ms) to eliminate any afterimages or lingering visual distractions. Subsequently, a fixation signal was displayed for 200 ms to centralize the participant’s gaze and stabilize their visual field in preparation for the stimulus. The target letter string was then presented at the center of the screen for 1,500 ms, allowing a uniform duration for participant responses. Post-response, the inter-trial interval (ITI) varied randomly between 3,000 to 7,000 ms prior to the onset of the next stimulus. This variable ITI was implemented to prevent participants from anticipating the timing of stimuli, ensuring that responses were spontaneous. Response times and accuracy were recorded by the E-prime software. These metrics are integral for analyzing the Stroop effect’s magnitude (MacLeod, 1991; Kane and Engle, 2003).

Prior to entering the MRI environment, we explained the task to the participants. Participants also practiced a short version of the same task outside of the MRI environment. The only differences from the MRI Stroop task was that participants were seated at a desk and used a keyboard to respond.

### 2.7 MRI acquisition

MRI data were collected using a 3 T Siemens Skyra scanner with a 32-channel head coil at the Center for Brain, Biology and Behavior at the University of Nebraska-Lincoln. A T1-weighted high resolution anatomical scan (TR = 2.2 s, TE = 3.37 ms, flip angle = 7°, FOV = 256 mm/100% phase, 192 slices, slice thickness = 1 mm) was collected for precise alignment of functional data. We also collected multiband echo-planar data (TR = 1 s, TE = 29.8 ms, flip angle = 60°, FOV = 210 mm/100% phase, slice thickness = 2.5 mm, 51 interleaved slices, multiband acceleration factor = 3) during in-scanner task performance (one run of 490 s prior to language learning and one run of 490 s following the language learning intervention).

### 2.8 fMRI preprocessing

Neuroimaging data were reconstructed using dcm2niix (Li et al., 2016). Following reconstruction, data preprocessing was completed using Analysis of Functional Neuroimages (AFNI) software (Cox, 1996). Code for the neuroimaging data processing and analysis is available at: https://github.com/dhschultz29/L2_learning_in_older_adults. Preprocessing consisted of despiking, slice time correction, non-linear transformation of anatomical data to MNI152_2009 template space, alignment of functional data to the transformed anatomical data, volume registration in which each volume is registered to the volume with the minimum outlier fraction, spatial smoothing using a 4 mm full-width at half maximum Gaussian filter, scaling the mean of each voxel’s time course to 100, and using a general linear model with three task regressors (correct congruent trials, correct incongruent trials, and correct neutral trials), 12 motion estimates (3 planes, 3 rotations, and their derivatives) from volume registration as regressors of no interest, and up to a sixth-order polynomial to model baseline and drift, all completed with the afni_proc.py function. We modeled hemodynamic response functions using the “BLOCK” basis function at the onset of each correct trial of each condition. We set the duration of the function to 1 s. Consecutive pairs of volumes where the Euclidean norm of the motion derivatives exceeded 0.4 were “scrubbed” and eliminated from analysis (Power et al., 2012) along with the first 4 TRs of the run. The mean beta weight for each condition was extracted for each participant, voxel, and time point for subsequent statistical analysis.

## 3 Data analysis

### 3.1 Behavioral data analysis

We calculated mean accuracy and reaction time data for congruent and incongruent trials for each participant. We ran an ANOVA with time (pre and post-language learning) and condition (congruent and incongruent) as repeated measures in JASP (ver. 0.16.2) (Love et al., 2019). The Holm-Bonferroni method was used for *post hoc* tests.

### 3.2 fMRI data analysis

We compared activation estimates for congruent and incongruent trials prior to the language learning intervention and following the language learning intervention using the 3dMVM function in AFNI (Chen et al., 2014). Participant was considered a random factor, condition and time point were modeled as within participant factors. We set a voxel-wise *p*-value threshold of 0.0001 for the main effects of condition (congruent/incongruent), time (pre-intervention, post-intervention), and the interaction of condition and time. We used a cluster-based approach to account for multiple comparisons for each of the main effects and the interaction (Forman et al., 1995). We estimated the smoothness of the residual time series and calculated the mean spatial autocorrelation parameters (ACF) across participants at pre-intervention (mean ACF = 0.7946, 2.1355, 4.8919) (Cox et al., 2017) and ten thousand random maps with these smoothness parameters were generated and thresholded at a voxelwise *p* < 0.001. The largest surviving cluster for each of these simulations was recorded and used to estimate the probability of a false positive. Based on these estimates we applied a cluster threshold to our data at a voxel-wise *p*-value of 0.001 and a minimum cluster size of seven voxels sharing either a face or an edge (NN = 2). This results in a corrected alpha of *p* < 0.05. Unthresholded statistical maps for the condition and time main effects, as well as the condition by time interaction can be found here: https://neurovault.org/collections/NLPFUQRB/.

## 4 Results

### 4.1 Adherence: exposure and use

Participants were encouraged to spend 60–90 min per day on language learning but were not restricted in the maximum amount of time they could spend. Given the importance of exposure and use on the neurocognitive effects of language learning (Ghazi Saidi et al., 2013, 2017; Ansaldo et al., 2015; Ghazi Saidi and Ansaldo, 2015, 2017a,b; Berroir et al., 2017; Carroll, 2017; Surrain and Luk, 2019; Olson, 2024), we monitored the amount of time that the participants used the online program. The mean cumulative time dedicated to the intervention by our participants was 4,885 min (min = 1,218; max = 14,522), with a standard deviation of 2,470 min, while the average time spent per day was 66.5 min, accompanied by a standard deviation of 25.1 min.

### 4.2 Proficiency

Each language program is divided into three levels, with participants required to take a proficiency test before advancing to the next level. We collected data on their test scores, which showed an average score of 96%. This indicates that all participants achieved a high proficiency level in the language lessons.

### 4.3 Behavioral results

**Figure 1:**
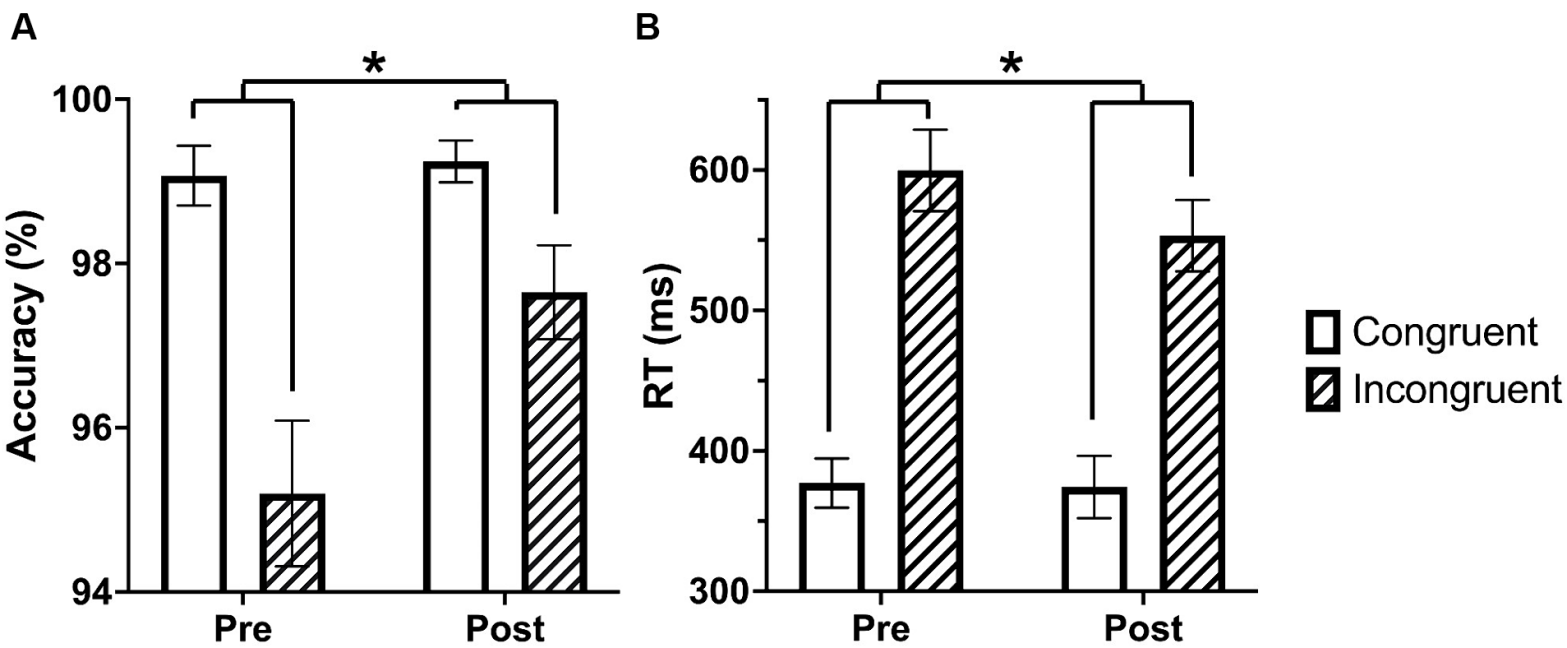
Performance on the Stroop task increases from pre to post language learning. **(A)** The difference between accuracy on congruent and incongruent trials decreases following language learning. **(B)** The difference between reaction time on congruent and incongruent trials decreases following language learning. Asterisks indicate interaction *p*-value <0.05.

An ANOVA with time (pre and post-language learning) and condition (congruent and incongruent) as repeated measures with the accuracy data identified a significant main effect for condition, *F* (1,40) = 25.373, *p* < 0.001, characterized by increased accuracy on congruent trials relative to incongruent trials. There was also a significant main effect for time, *F*(1,40) = 5.219, *p* = 0.028, characterized by more accurate performance after language learning relative to before. We also found a significant condition by time interaction, *F*(1,40) = 8.203, *p* = 0.007, characterized by a greater difference in accuracy between congruent and incongruent trials before, *t* = 5.756, *p* < 0.001, and less difference in accuracy between congruent and incongruent trials after language learning, *t* = 2.37, *p* = 0.061 (Figure 1A).

An ANOVA with time (pre and post-language learning) and condition (congruent and incongruent) as repeated measures with the reaction time data identified a significant main effect for condition, *F*(1,40) = 218.813, *p* < 0.001, characterized by faster reaction time on congruent trials relative to incongruent trials. We also found a significant condition by time interaction, *F*(1,40) = 10.010, *p* = 0.003, characterized by a difference in reaction time between incongruent trials pre-language learning and post-language learning with faster reaction time post-language learning, *t* = 3.213, *p* = 0.004, and no difference in reaction time between pre-and post-language learning on congruent trials, *t* = 0.192, *p* = 0.849. There was not a significant main effect for time, *F*(1,40) = 3.752, *p* = 0.06, (Figure 1B).

### 4.4 fMRI results

#### 4.4.1 Stroop main effect

**Figure 2:**
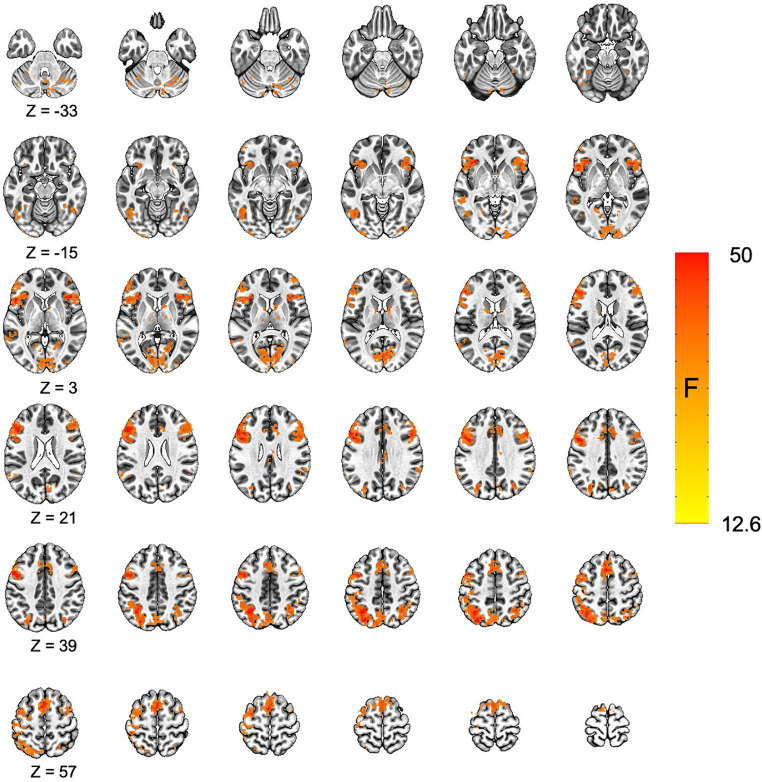
Increased activation for incongruent relative to congruent trials in the Stroop task. The main effect for condition (incongruent vs. congruent) is displayed. All significant results were characterized by greater activation for the incongruent relative to congruent condition. Cluster corrected *p*-value <0.05.

Voxel-wise analysis of the fMRI data was conducted using 3dMVM in AFNI. The multivariate modeling approach identified a main effect for condition (incongruent vs. congruent trials). All significant clusters were characterized by greater responses on incongruent trials relative to congruent trials. We identified significant differences between incongruent and congruent trials in portions of the lateral prefrontal cortex, parietal cortex, and the anterior cingulate, consistent with previous studies using the Stroop task (Song and Hakoda, 2015; Huang et al., 2020; Mill et al., 2020; Figure 2 and Table 1).

**Table 1:** Significant activation clusters from the Stroop task.

					MNI Coordinates (CM)		
Structures	Direction	Hemisphere	*F*-statistic	Volume (mm^3)	*X*	*Y*	*Z*
Inferior frontal gyrus	I > C	Left	36.71	27,453	−47	17	25
	I > C	Right	32.23	14,860	47	22	18
	I > C	Right	19.36	250	49	44	−4
Middle frontal gyrus	I > C	Right	12.7	1,109	42	52	9
	I > C	Right	17.46	125	37	38	29
	I > C	Left	14.83	188	−38	50	25
Precentral gyrus	I > C	Left	28.1	203	−27	−10	56
SMA	I > C	Both	47.97	14,267	−1	14	53
Superior frontal gyrus	I > C	Right	15.47	172	28	1	57
Angular gyrus	I > C	Right	21.73	4,625	35	−61	46
Calcarine gyrus	I > C	Both	13.18	12,345	3	−81	8
Inferior parietal lobe	I > C	Left	46.49	21,189	−30	−60	49
	I > C	Right	14.32	125	54	−51	26
Supramarginal gyrus	I > C	Right	17.33	406	60	−44	30
	I > C	Left	16.1	234	−64	−48	34
Cuneus	I > C	Right	24	172	7	−79	35
Fusiform gyrus	I > C	Right	23	234	33	−49	−17
	I > C	Right	16.25	172	32	−58	−12
	I > C	Left	18.29	125	−33	−49	−16
Inferior temporal gyrus	I > C	Left	13.77	594	−31	−98	−10
	I > C	Right	14.94	234	41	−88	−6
Middle occipital gyrus	I > C	Right	20.88	219	37	−88	4
Inferior temporal gyrus	I > C	Left	23.45	2,516	−48	−63	−9
	I > C	Right	20.35	203	48	−69	−11
	I > C	Right	34.03	188	49	−56	−14
Middle temporal gyrus	I > C	Left	18.66	2,047	−59	−48	7
	I > C	Left	18.97	219	−51	−53	19
Middle cingulate	I > C	Both	14.32	313	−1	−28	29
	I > C	Right	19.89	141	7	−14	31
Caudate nucleus	I > C	Right	27.6	938	11	1	9
	I > C	Left	17.37	328	−12	1	13
Cerebellum	I > C	Right	25.28	2,281	17	−71	−30
	I > C	Left	28.43	234	−47	−68	−29
	I > C	Left	14.24	203	−9	−82	−24
	I > C	Left	13.29	156	−31	−57	−29
	I > C	Right	13.1	125	36	−73	−27
Thalamus	1 > C	Left	31.03	156	−12	−16	9

*F*-statistic reflects value at center of mass or nearest voxel to the center of mass. I > C indicates incongruent was greater than congruent.

#### 4.4.2 Time main effect and condition by time interaction

We did not observe any significant clusters for the main effect of time (pre vs. post-language learning), and we did not find any significant clusters for the condition (incongruent and congruent) by time (pre and post-language learning) interaction.

#### 4.4.3 Time spent with the language learning program is related to changes in activity during Stroop task

The literature on bilingualism (Carroll, 2017; Surrain and Luk, 2019; Olson, 2024), and our previous neuroimaging study results (Ghazi Saidi et al., 2013, 2017; Ansaldo et al., 2015; Ghazi Saidi & Ansaldo, 2015, 2017a,b; Berroir et al., 2017) suggest that three main factors impact cognitive processes in bilingual speakers: use, exposure, and proficiency. The results on participants’ performance in this study indicated that all participants achieved a high proficiency level (an average of % 96 on all proficiency tests). However, the time spent on the language learning program, reflecting exposure and use, varied among participants (min = 1,218; max = 14,522; *M* = 4,885; SD = 2,470 min). Although within the design of this study it is not possible to tease apart exposure and use, we used the cumulative time tracked by the language learning software, as a variable that reflects exposure and use combined.

**Figure 3:**
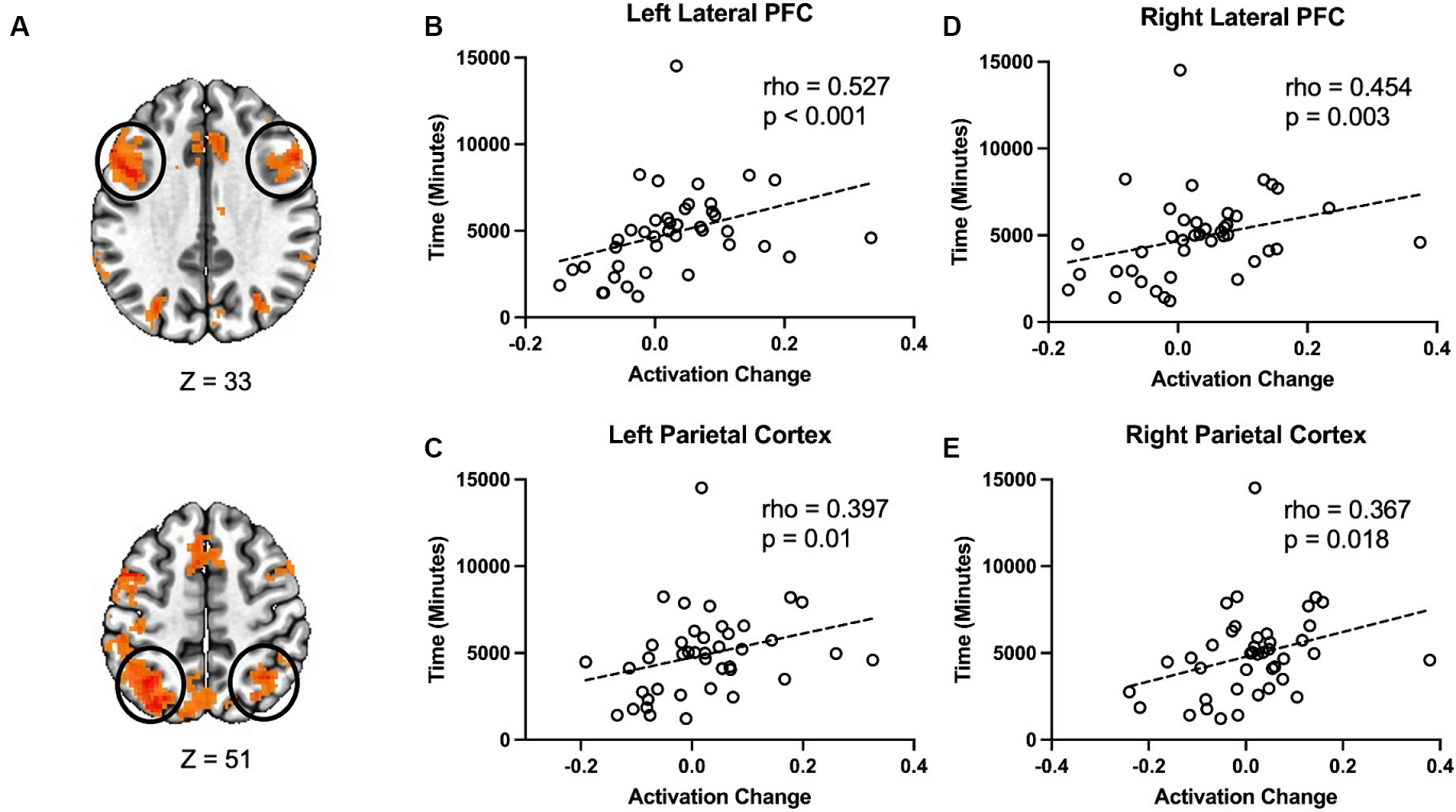
Changes in Stroop activation are related to the amount of time spent on the language learning program. **(A)** Changes in Stroop task activation were extracted from four clusters identified in the main effect for condition. These clusters were selected based on their involvement in the Stroop task in previous studies as well as from the main effect of condition in the current study. Correlation between the change in Stroop activity and the time participants spent with the language learning program in the **(B)** Left lateral prefrontal cortex, **(C)** Left parietal cortex, **(D)** Right lateral prefrontal cortex, and **(E)** Right parietal cortex.

Therefore, we examined the possibility of whether the amount of time each participant spent using the language learning program had an effect on the changes in task-evoked activity from baseline to after the language learning intervention. First, we calculated a metric of change in activation over time. We calculated the difference score between incongruent and congruent conditions for each time point. Then we subtracted the pre-difference score from the post-language learning difference score [(post incongruent – post congruent) – (pre incongruent – pre congruent)]. The result is a measure where higher values reflect a greater difference between incongruent and congruent post-language learning relative to pre. This measure was calculated for four regions (right and left lateral prefrontal cortex and right and left parietal cortex, see Figure 3A) that showed a main effect for task condition, and which have been previously implicated in performance during the Stroop task. Finally, we used Spearman rank correlation to examine the relationship between changes in activation from pre to post-language learning and the total amount of time each participant spent using the language learning program. The change in activation was positively correlated with time spent on the language learning program, *rho* = 0.527, *p* < 0.001 (Figure 3B), in the left prefrontal cortex and the left parietal cortex, *rho* = 0.397, *p* = 0.01 (Figure 3C). There was also a positive relationship between changes in activity and time spent on the language learning program in the right lateral prefrontal cortex, *rho* = 0.454, *p = 0.003,* (Figure 3D) and the right parietal cortex, *rho* = 0.367, *p* = 0.018 (Figure 3E).

We observed that changes in Stroop activation were related to the amount of time people spent on the language learning program. Next we examined whether time spent on the language learning program was related to the change in performance on the task. Based on the fMRI results, we hypothesized that increases in behavioral performance would be positively correlated with the amount of time spent on the language learning program. We calculated a behavioral change score for both accuracy and reaction time. This behavioral change score followed the same logic as that used for the fMRI activation change score. However, as improvements in performance over time result in more similar accuracy and reaction time for congruent and incongruent times (see Figure 1), we reversed the subtraction operations so that more positive scores could be interpreted as increases in performance from pre to post intervention for both accuracy and reaction time. Therefore, the formula for accuracy was [(pre congruent – pre incongruent) – (post congruent – post incongruent)]. Additionally, there was greater between-participant variability in reaction times, so to account for this variability we calculated the change score as a proportion of the difference between incongruent and congruent trials pre-intervention. Thus, the formula for reaction time was [(pre incongruent – pre congruent) – (post incongruent – post congruent)/(pre incongruent – pre congruent)]. Next, we correlated the behavioral change scores with the amount of time spent on the language learning program. As our hypothesis was that this relationship would be positive, we used a one-tailed test. We did not observe a significant relationship between time spent on the language learning program and improvements in either accuracy or reaction time on the Stroop task, *smallest one-tailed p* = 0.199.

While we did not observe a significant linear relationship between time spent on the language learning program and improvements in Stroop performance, we split participants into two groups, those who spent more time with the language learning program and those who spent less time. Participants were placed in these groups if they spent more or less than the mean amount of time with the language learning program (4,885 min or ~ 81.5 h). Then we evaluated whether or not these groups were characterized by different levels of improvement on Stroop task performance (as described above). We found that the group who spent more time with the language learning program showed greater increases in Stroop accuracy from pre to post-intervention, *t*(39) = 1.846, *one-tailed p* = 0.036. We did not observe any differences in reaction time performance between the groups who spent more or less time with the language learning program, *t*(39) = −1.036*, one-tailed p* = 0.153. These data suggest that time spent with the language learning program was related to greater accuracy gains from pre to post-intervention on the Stroop. While the effect on reaction time was not significant, it was in the opposite direction as the accuracy effect which may be suggestive of a speed/accuracy trade off.

## 5 Discussion

We hypothesized that learning a new language could serve as a beneficial intervention to boost cognitive performance for older adults. By leveraging the cognitive complexity inherent in acquiring a new language, we hypothesized that L2 learning would lead to specific cognitive domains such as executive functions, such as enhanced cognitive control, and quicker information processing.

We observed a significant condition (congruent vs. incongruent) by time (pre and post-language learning) interactions on measures of accuracy and reaction time for the Stroop task. These interactions were characterized by an increase in accuracy and a decrease in reaction time on incongruent trials post intervention. Changes in performance on incongruent trials, which require conflict management (e.g., “red” written in green ink), reflect enhanced executive functioning. In contrast, congruent trials, which do not involve conflict (e.g., “red” written in red ink), showed no significant change from pre to post-language learning. This suggests the changes observed on incongruent trials are not general practice effects; but related to the intervention.

Behavioral results showed the Stroop effects and an improved performance post intervention as compared to pre intervention. Specifically, pre-post intervention comparisons reflected that the performance in Stroop task was more accurate post intervention as compared to pre intervention and the accuracy of the incongruent trials, which require more cognitive control, were improved and closer to the easier congruent trials which are less demanding in terms of conflict management. Response times showed a similar effect to accuracy suggesting language learning influenced processing speed as well. Together, these data support the idea that Stroop performance improves following language learning.

The functional neuroimaging results support the behavioral results. The activation maps regarding contrasts between incongruent and congruent trials are consistent with previous studies using the Stroop task (Song and Hakoda, 2015; Huang et al., 2020; Mill et al., 2020), with greater activations for the incongruent trials reflecting higher cognitive demand required for processing incongruent trials. The significant activations observed were in parts of the lateral prefrontal cortex, parietal cortex, and the anterior cingulate, all of which are involved in executive function, inhibitory control, conflict management and working memory (De Pisapia et al., 2006; Ardila, 2019; Friedman and Robbins, 2022), cognitive domains required for processing the Stroop task (Ye and Zhou, 2009; Heidlmayr et al., 2020).

While we did not observe any significant effect of time (pre vs. post-language learning), or for the interaction (condition by time), we did observe a relationship between changes in the brain activity during Stroop task and the amount of time participants spent on language learning. Specifically, we found a positive relationship between changes in brain activity and time spent on the language learning program in the right lateral prefrontal cortex, the left prefrontal cortex and the left parietal cortex, as well as the right lateral prefrontal cortex. This suggests that the time spent on learning a new language can modulate activation related to the Stroop task in regions included in the frontoparietal network (FPN).

The FPN and cingulate play major roles in the organization of executive function, including working memory, conflict management, inhibitory control, and planning (Friedman and Robbins, 2022; Menon and D’Esposito, 2022). The FPN, with its flexible hubs, specially in the right hemisphere, is instrumental in modulating activity across various distributed systems, such as visual, limbic, and motor networks, to align with goals of task in hand, directly supporting working memory and task-switching capabilities (Cole et al., 2014). On the other hand, the cingulo-opercular network (CON) is crucial for maintaining task sets, enabling sustained cognitive performance across different tasks (Hausman et al., 2022). The CON functions through connections with other hub regions, including the anterior cingulate and anterior insula, which are associated with better performance in working memory, inhibition, and set-shifting tasks (Bush and Shin, 2006; Hausman et al., 2022). Together, these networks form part of a superordinate cognitive control system that includes the dorsolateral prefrontal, anterior cingulate, and parietal cortices, underpinning a broad array of executive functions (Niendam et al., 2012). Patterns of activity in regions of these networks can contain information about diverse task rules across multiple domains illustrating how they may function to orchestrate goal-directed behavior (Ito et al., 2017; Schultz et al., 2022). This superordinate network can describe how the executive function system with a common infrastructure can cater to the requirements of a network-specific and domain-specific demands of a particular task.

Both dorsolateral prefrontal and parietal cortex have been consistently reported as key brain areas associated with Stroop task performance that are involved in cognitive control and interference control (Huang et al., 2020). Dorsolateral prefrontal and parietal cortex are consistently implicated in Alzheimer’s disease and mild cognitive impairment (Morbelli et al., 2013; Giovacchini et al., 2019), and as critical brain areas related to cognitive reserve (Ye and Zhou, 2009; Dodich et al., 2018; Giovacchini et al., 2019). Interestingly, these brain areas have been reported as important brain areas that contribute to the cognitive advantage in lifelong bilinguals as compared to monolingual peers. The current results suggest that learning a new language in older adults for 4 months may improve Stroop performance. These results may also suggest that learning a new language in older adults can contribute to improving cognitive reserve and may contribute to postponing or slowing cognitive decline.

These results are consistent with studies that have explored a similar research question. Specifically, to date, there are four studies that have explored the cognitive effect of language learning in older adults (Bubbico et al., 2019; Wong et al., 2019; Nilsson et al., 2021; Meltzer et al., 2023). These studies use different measures and therefore the results are not easy to compare. Nevertheless, all four studies evidence some improvement post intervention (language learning) in a specific cognitive domain (Nilsson et al., 2021; Meltzer et al., 2023) or global cognition (Bubbico et al., 2019; Wong et al., 2019). Meltzer et al. (2023) reported a behavioral study in 76 older adults aged 65–75, similar to the results of our study, and found that Stroop accuracy improved after 16 weeks of either language learning with Duolingo or with an equivalent amount of non-language cognitive training. These results are consistent with what we observed in the current study. However, Meltzer et al. (2023) report that the non-language cognitive training condition resulted in improvements in reaction time on the Stroop while Duolingo did not. These results are in contrast to the current study where we did observe an effect of language learning on reaction time on the Stroop task. It is unclear whether potential differences in the dose, duration, intensity of the intervention, or an additional factor, may contribute to this difference.

Another study examined the neurocognitive effects of four-month second language learning program on the brain function of healthy elderly individuals (age 59–79) in a behavioral and neuroimaging study (Bubbico et al., 2019). They reported the behavioral impact as limited to global cognition. However, post-program, participants showed increased resting state functional connectivity in the right inferior frontal gyrus, right superior frontal gyrus, and left superior parietal lobule. Collectively, these brain areas are involved in numerous processes including: inhibitory control, decision making, attentional control, processing indirect language components such as emotional aspects, (Simonet, 2014; Eriksen and Zacharov, 2016), maintaining and manipulating information in working memory, as well as in directing attention (Manelis and Reder, 2014), and spatial attention and orientation (Heinen et al., 2017). In the current study, the contrast between incongruent and congruent trials on the Stroop task yielded activation of left lateral prefrontal cortex, and right lateral prefrontal cortex, as well as the left and the right parietal cortex. Although not a precise overlap, the activation map we observed is consistent with many of the regions showing changes in resting-state connectivity by Bubbico et al. (2019).

Altogether, in line with the previous reports (Bubbico et al., 2019; Meltzer et al., 2023), we provide evidence that even short-term language learning interventions can significantly reorganize and enhance neural network functions in older adults, contributing to better cognitive performance in specific executive function tasks such as the Stroop task. It is important, however, to note that while Stroop performance improved from pre to post-intervention and brain activity was modulated by the amount of time people spent with the language learning program, the dose dependent effects of time spent on the language learning program and improvements in Stroop performance were tenuous. Future studies should determine the optimal intensity, frequency, duration and intensity of language learning programs as an effective cognitive intervention in older adults.

We argue that language learning with sufficient dose, intensity and frequency can be considered as an effective cognitive intervention for promoting healthy aging. Based on our understanding of cognitive reserve (Stern, 2002, 2009, 2021; Stern et al., 2020; Nelson et al., 2021) and the factors that can contribute to strengthening cognitive reserve (Stern et al., 2020), this intervention may potentially counteract the cognitive decline associated with aging through the enhancement of brain plasticity and functional connectivity. The latter hypothesis remains to be tested.

As a side note, our results provide further evidence to recognize the Stroop test as a valuable tool for assessing cognitive health in aging populations. The Stroop test has been recommended for early detection of cognitive impairment in individuals with subjective cognitive complaints (Gajewski et al., 2020; Rao et al., 2023); as well as for differentiating individuals with mild Alzheimer’s disease (AD) from non-AD individuals, and to distinguish between mild and severe AD (Lin and Lai, 2024).

## 6 Conclusion

We bring evidence that language learning at older ages may boost cognitive control performance, as measured by improvements in the Stroop task. This enhancement is associated with functional neuroplasticity in cognitive control areas of the brain, indicating that acquiring a new language may actively influence function in these crucial regions. Specifically, these changes are observable improvements in tasks that require attentional control, such as the Stroop task, reflecting the transfer of cognitive gains from language learning to other cognitive domains. These results align with the theory of cognitive reserve, suggesting that intellectually stimulating activities like language learning can bolster the brain’s resilience to age-related decline. However, we also acknowledge that the extent of these benefits depends on the dose of language engagement, highlighting the importance of the amount and intensity of learning in realizing cognitive advantages. These results present a promising avenue for non-pharmaceutical intervention for aging related cognitive decline, offering an accessible, low cost and potentially enjoyable approach to maintaining and enhancing cognitive health in the aging population. As populations globally are experiencing increased longevity, understanding how to maintain cognitive health in later life is of paramount importance.

### 6.1 Limitations and future research

Our study has a few limitations. First, our study’s design involved 41 participants, and is likely too small to allow for broad generalizations. In addition, Dichotomization of data may increase the risk of obtaining a false positive result (Altman and Royston, 2006). Second, our study employed a pre-post intervention design without incorporating a control group or control condition, limiting our ability to isolate the intervention effect from other potential influencing factors such as practice effects. In addition, a lack of control groups limits drawing a strong conclusion for the causality of the intervention effects, including a placebo effect, or controlling for confounding factors such as interaction with the researchers or the logistics of the language program such as logging in and out, and following instructions, as well as limiting the generalizability of our findings. The challenges associated with conducting randomized trials on second language acquisition as a cognitive intervention in older adults, have limited research in this area. Future research approaches with bigger samples and designs that include control conditions are required. Third, this study potentially exhibits self-selection sample bias, as the personality types of the participants and demographic homogeneity could have influenced the composition of our sample. The participants were homogeneously white Caucasian, which is reflective of the population characteristics of Nebraska. Future multi-site studies should include all races and different ethnicities. In addition, our sample consisted of older adults with moderate to high education levels (M = 17.5, SD = 2.94). Higher education level may have impacted the learning process or the cognitive demand required for learning a new language, and thus the results. Future studies should explore the effect of education level on the cognitive effect of language learning. Further, the ethnic minorities were excluded due to their proficiency in two or more languages. Future research should look at the cognitive effects of a third language in bilinguals of different ethnicities. Finally, our priority was to allow participants to select the language of their choice. This approach does not account for the effect of language distance. Future studies should explore the language distance effect. Thus, comprehensive research on cognitive effects of language learning is required to support our evidence for cognitive benefits of learning a new language in older adults. Further research can provide invaluable insights into cognitive aging, offering strategies not only for individual cognitive health but also for societal well-being.

## Data availability statement

The original contributions presented in the study are included in the article/supplementary materials, further inquiries can be directed to the corresponding author.

## Ethics Statement

The studies involving humans were approved by Rachel Wenzl - University of Nebraska-Lincoln. The studies were conducted in accordance with the local legislation and institutional requirements. The participants provided their written informed consent to participate in this study.

## Author contributions

DS: Data curation, Formal analysis, Funding acquisition, Investigation, Methodology, Visualization, Writing – original draft, Writing – review & editing. AG: Investigation, Writing – review & editing. KA: Investigation, Writing – review & editing. MG: Investigation, Writing – review & editing. LS: Investigation, Writing – review & editing. ZD: Resources, Writing – review & editing. CS: Funding acquisition, Writing – review & editing. LG: Conceptualization, Data curation, Funding acquisition, Investigation, Methodology, Project administration, Resources, Supervision, Writing – original draft, Writing – review & editing.

## Footnotes

^1^ https://www.rosettastone.com/

## References

[ref1] Abutalebi J. Green D. (2007). Bilingual language production: The neurocognition of language representation and control. J. Neurolinguistics 20, 242–275. doi: 10.1016/j.jneuroling.2006.10.003

[ref2] Altman D. G. Royston P. (2006). The cost of dichotomising continuous variables. BMJ 332:1080. doi: 10.1136/bmj.332.7549.1080, PMID: 16675816 PMC1458573

[ref3] Ansaldo A. I. Ghazi-Saidi L. Adrover-Roig D. (2015). Interference control in elderly bilinguals: Appearances can be misleading. J. Clin. Exp. Neuropsychol. 37, 455–470. doi: 10.1080/13803395.2014.990359, PMID: 25641572

[ref4] Antoniou M. (2019). The advantages of bilingualism debate. Ann. Rev. Linguist. 5, 395–415. doi: 10.1146/annurev-linguistics-011718-011820, PMID: 38714635

[ref5] Antoniou M. Gunasekera G. M. Wong P. C. M. (2013). Foreign language training as cognitive therapy for age-related cognitive decline: a hypothesis for future research. Neurosci. Biobehav. Rev. 37, 2689–2698. doi: 10.1016/j.neubiorev.2013.09.004, PMID: 24051310 PMC3890428

[ref6] Ardila A. (2019). “Executive functions brain functional system” in *Dysexecutive Syndromes*. eds. Ardila A. Fatima S. Rosselli M. (Switzerland AG: Springer International Publishing), 29–41. doi: 10.1007/978-3-030-25077-5_2

[ref7] Barbu C. Orban S. Gillet S. Poncelet M. (2018). The Impact of Language Switching Frequency on Attentional and Executive Functioning in Proficient Bilingual Adults. Psychol. Belgica 58, 115–127. doi: 10.5334/pb.392, PMID: 30479811 PMC6194534

[ref8] Bari A. Robbins T. W. (2013). Inhibition and impulsivity: Behavioral and neural basis of response control. Prog. Neurobiol. 108, 44–79. doi: 10.1016/j.pneurobio.2013.06.005, PMID: 23856628

[ref9] Berroir P. Ghazi-Saidi L. Dash T. Adrover-Roig D. Benali H. Ansaldo A. I. (2017). Interference control at the response level: Functional networks reveal higher efficiency in the bilingual brain. J. Neurolinguistics 43, 4–16. doi: 10.1016/j.jneuroling.2016.09.007

[ref10] Bialystok E. (2021). Bilingualism: Pathway to Cognitive Reserve. Trends Cogn. Sci. 25, 355–364. doi: 10.1016/j.tics.2021.02.003, PMID: 33771449 PMC8035279

[ref11] Bialystok E. Abutalebi J. Bak T. H. Burke D. M. Kroll J. F. (2016). Aging in two languages: Implications for public health. Ageing Res. Rev. 27, 56–60. doi: 10.1016/j.arr.2016.03.003, PMID: 26993154 PMC4837064

[ref12] Bialystok E. Craik F. I. (2022). How does bilingualism modify cognitive function? Attention to the mechanism. Psychon. Bull. Rev. 29, 1246–1269. doi: 10.3758/s13423-022-02057-5 35091993

[ref13] Bialystok E. Craik F. I. M. Freedman M. (2007). Bilingualism as a protection against the onset of symptoms of dementia. Neuropsychologia 45, 459–464. doi: 10.1016/j.neuropsychologia.2006.10.009, PMID: 17125807

[ref14] Bialystok E. Craik F. I. Freedman M. (2024). 7 Bilingualism as a protection against the onset of symptoms of dementia. Where language meets thought: selected works of Ellen Bialystok, 221. 10.1016/j.neuropsychologia.2006.10.009 17125807

[ref15] Bott H. Madero G. Fuseya G. Gray M. (2019). Face-to-Face and Digital Multidomain Lifestyle Interventions to Enhance Cognitive Reserve and Reduce Risk of Alzheimer’s Disease and Related Dementias: A Review of Completed and Prospective Studies. Nutrients 11:2258. doi: 10.3390/nu11092258, PMID: 31546966 PMC6770494

[ref16] Brown C. A. Weisman de Mamani A. (2017). A comparison of psychiatric symptom severity in individuals assessed in their mother tongue versus an acquired language: A two-sample study of individuals with schizophrenia and a normative population. Prof. Psychol. Res. Pract. 48:1. doi: 10.1037/pro0000125

[ref17] Bubbico G. Chiacchiaretta P. Parenti M. di Marco M. Panara V. Sepede G.. (2019). Effects of Second Language Learning on the Plastic Aging Brain: Functional Connectivity, Cognitive Decline, and Reorganization. Front. Neurosci. 13:423. doi: 10.3389/fnins.2019.00423, PMID: 31156360 PMC6529595

[ref18] Bush G. Shin L. M. (2006). The Multi-Source Interference Task: An fMRI task that reliably activates the cingulo-frontal-parietal cognitive/attention network. Nat. Protoc. 1, 308–313. doi: 10.1038/nprot.2006.48, PMID: 17406250

[ref19] Cammisuli D. M. Franzoni F. Scarfò G. Fusi J. Gesi M. Bonuccelli U.. (2022). What does the brain have to keep working at its best? Resilience mechanisms such as antioxidants and brain/cognitive reserve for counteracting Alzheimer’s disease degeneration. Biology 11:650. doi: 10.3390/biology11050650, PMID: 35625381 PMC9138251

[ref20] Carroll S. E. (2017). Exposure and input in bilingual development. Biling. Lang. Congn. 20, 3–16. doi: 10.1017/S1366728915000863, PMID: 38714153

[ref21] Chen G. Adleman N. E. Saad Z. S. Leibenluft E. Cox R. W. (2014). Applications of multivariate modeling to neuroimaging group analysis: A comprehensive alternative to univariate general linear model. NeuroImage 99, 571–588. doi: 10.1016/j.neuroimage.2014.06.027, PMID: 24954281 PMC4121851

[ref22] Cole M. W. Repovš G. Anticevic A. (2014). The Frontoparietal Control System: A Central Role in Mental Health. Neuroscientist 20, 652–664. doi: 10.1177/1073858414525995, PMID: 24622818 PMC4162869

[ref23] Costumero V. Rodríguez-Pujadas A. Fuentes-Claramonte P. Ávila C. (2015). How bilingualism shapes the functional architecture of the brain: a study on executive control in early bilinguals and monolinguals. Hum. Brain Mapp. 36, 5101–5112. doi: 10.1002/hbm.22996, PMID: 26376449 PMC6869221

[ref24] Cox R. W. (1996). AFNI: Software for analysis and visualization of functional magnetic resonance neuroimages. Comput. Biomed. Res. 29, 162–173. doi: 10.1006/cbmr.1996.0014, PMID: 8812068

[ref25] Cox R. W. Chen G. Glen D. R. Reynolds R. C. Taylor P. A. (2017). fMRI clustering and false-positive rates. Proc. Natl. Acad. Sci. 114, E3370–E3371. doi: 10.1073/pnas.1614961114, PMID: 28420798 PMC5410825

[ref26] Craik F. I. M. Bialystok E. Freedman M. (2010). Delaying the onset of Alzheimer disease: Bilingualism as a form of cognitive reserve. Neurology 75, 1726–1729. doi: 10.1212/WNL.0b013e3181fc2a1c, PMID: 21060095 PMC3033609

[ref27] De Pisapia N. Slomski J. A. Braver T. S. (2006). Functional Specializations in Lateral Prefrontal Cortex Associated with the Integration and Segregation of Information in Working Memory. Cereb. Cortex 17, 993–1006. doi: 10.1093/cercor/bhl010, PMID: 16769743

[ref28] Degirmenci M. G. Grossmann J. A. Meyer P. Teichmann B. (2022). The role of bilingualism in executive functions in healthy older adults: a systematic review. Int. J. Biling. 26, 426–449. doi: 10.1177/13670069211051291

[ref29] Del Maschio N. Sulpizio S. Gallo F. Fedeli D. Weekes B. S. Abutalebi J. (2018). Neuroplasticity across the lifespan and aging effects in bilinguals and monolinguals. Brain Cogn. 125, 118–126. doi: 10.1016/j.bandc.2018.06.007, PMID: 29990701

[ref30] Di Nuovo S. De Beni R. Borella E. Marková H. Laczó J. Vyhnálek M. (2020). Cognitive impairment in old age: is the shift from healthy to pathological aging responsive to prevention? Eur. Psychol. 25, 174–185. doi: 10.1027/1016-9040/a000391

[ref31] Dodich A. Carli G. Cerami C. Iannaccone S. Magnani G. Perani D. (2018). Social and cognitive control skills in long-life occupation activities modulate the brain reserve in the behavioural variant of frontotemporal dementia. Cortex 99, 311–318. doi: 10.1016/j.cortex.2017.12.006, PMID: 29328983

[ref32] Dyer S. M. Harrison S. L. Laver K. Whitehead C. Crotty M. (2018). An overview of systematic reviews of pharmacological and non-pharmacological interventions for the treatment of behavioral and psychological symptoms of dementia. Int. Psychogeriatr. 30, 295–309. doi: 10.1017/S1041610217002344, PMID: 29143695

[ref33] Egner T. Hirsch J. (2005). The neural correlates and functional integration of cognitive control in a Stroop task. NeuroImage 24, 539–547. doi: 10.1016/j.neuroimage.2004.09.007, PMID: 15627596

[ref34] Eriksen K. Zacharov O. (2016). Investigating the role of the right inferior frontal gyrus in response inhibition: an fMRI approach. Master in Cognitive Neuroscience Department of Psychology. Available at: https://www.duo.uio.no/bitstream/handle/10852/51234/1/FINAL.pdf

[ref35] Forman S. D. Cohen J. D. Fitzgerald M. Eddy W. F. Mintun M. A. Noll D. C. (1995). Improved assessment of significant activation in functional magnetic resonance imaging (fMRI): Use of a cluster-size threshold. Magn. Reson. Med. 33, 636–647. doi: 10.1002/mrm.1910330508, PMID: 7596267

[ref36] Friedman N. P. Robbins T. W. (2022). The role of prefrontal cortex in cognitive control and executive function. Neuropsychopharmacology 47, 72–89. doi: 10.1038/s41386-021-01132-0, PMID: 34408280 PMC8617292

[ref37] Fukuta J. Yamashita J. (2015). Effects of cognitive demands on attention orientation in L2 oral production. System 53, 1–12. doi: 10.1016/j.system.2015.06.010

[ref38] Gajewski P. D. Falkenstein M. Thönes S. Wascher E. (2020). Stroop task performance across the lifespan: High cognitive reserve in older age is associated with enhanced proactive and reactive interference control. NeuroImage 207:116430. doi: 10.1016/j.neuroimage.2019.116430, PMID: 31805383

[ref39] Gallo F. Abutalebi J. (2024). The unique role of bilingualism among cognitive reserve-enhancing factors. Biling. Lang. Congn. 27, 287–294. doi: 10.1017/S1366728923000317

[ref40] García-Pentón L. Pérez Fernández A. Iturria-Medina Y. Gillon-Dowens M. Carreiras M. (2014). Anatomical connectivity changes in the bilingual brain. NeuroImage 84, 495–504. doi: 10.1016/j.neuroimage.2013.08.064, PMID: 24018306

[ref41] Ghazi Saidi L. G. Ansaldo A. I. (2015). Can a second language help you in more ways than one. Aims Neurosci. 2, 52–57. doi: 10.3934/Neuroscience.2015.1.52

[ref42] Ghazi Saidi L. G. Dash T. Ansaldo A. I. (2017). The bilingual mental lexicon: a dynamic knowledge system. In Bilingualism 73–102. John Benjamins.

[ref43] Ghazi Saidi L. G. Perlbarg V. Marrelec G. Pélégrini-Issac M. Benali H. Ansaldo A. I. (2013). Functional connectivity changes in second language vocabulary learning. Brain Lang. 124, 56–65. doi: 10.1016/j.bandl.2012.11.008, PMID: 23274799

[ref44] Ghazi-Saidi L. Ansaldo A. I. (2017b). The neural correlates of semantic and phonological transfer effects: Language distance matters. Biling. Lang. Congn. 20, 1080–1094. doi: 10.1017/S136672891600064X

[ref45] Ghazi-Saidi L. Ansaldo A. I. (2017a). Second language word learning through repetition and imitation: Functional networks as a function of learning phase and language distance. Front. Hum. Neurosci. 11:277215. doi: 10.3389/fnhum.2017.00463, PMID: 29033804 PMC5625023

[ref47] Giovacchini G. Giovannini E. Borsò E. Lazzeri P. Riondato M. Leoncini R.. (2019). The brain cognitive reserve hypothesis: A review with emphasis on the contribution of nuclear medicine neuroimaging techniques. J. Cell. Physiol. 234, 14865–14872. doi: 10.1002/jcp.28308, PMID: 30784080

[ref48] Green D. W. Abutalebi J. (2013). Language control in bilinguals: The adaptive control hypothesis. J. Cogn. Psychol. 25, 515–530. doi: 10.1080/20445911.2013.796377, PMID: 25077013 PMC4095950

[ref49] Guarino A. Forte G. Giovannoli J. Casagrande M. (2020). Executive functions in the elderly with mild cognitive impairment: A systematic review on motor and cognitive inhibition, conflict control and cognitive flexibility. Aging Ment. Health 24, 1028–1045. doi: 10.1080/13607863.2019.1584785, PMID: 30938193

[ref50] Guo T. Ma F. (2023). “Cognitive control in second language neurocognition” in The Routledge Handbook of Second Language Acquisition and Neurolinguistics. eds. K. Morgan-Short and J. G. van Hell (New York: Routledge), 424–435.

[ref51] Han X. Li W. Filippi R. (2024). Modulating bilingual language production and cognitive control: how bilingual language experience matters. Bilingualism Lang. Cognit. 1–15. doi: 10.1017/S1366728924000191

[ref52] Hausman H. K. Hardcastle C. Albizu A. Kraft J. N. Evangelista N. D. Boutzoukas E. M.. (2022). Cingulo-opercular and frontoparietal control network connectivity and executive functioning in older adults. Gero Sci. 44, 847–866. doi: 10.1007/s11357-021-00503-1, PMID: 34950997 PMC9135913

[ref53] Heidlmayr K. Kihlstedt M. Isel F. (2020). A review on the electroencephalography markers of Stroop executive control processes. Brain Cogn. 146:105637. doi: 10.1016/j.bandc.2020.105637, PMID: 33217721

[ref54] Heinen K. Feredoes E. Ruff C. C. Driver J. (2017). Functional connectivity between prefrontal and parietal cortex drives visuo-spatial attention shifts. Neuropsychologia 99, 81–91. doi: 10.1016/j.neuropsychologia.2017.02.024, PMID: 28254653 PMC5415819

[ref55] Hertrich I. Dietrich S. Blum C. Ackermann H. (2021). The role of the dorsolateral prefrontal cortex for speech and language processing. Front. Hum. Neurosci. 15:645209. doi: 10.3389/fnhum.2021.645209, PMID: 34079444 PMC8165195

[ref56] Hilchey M. D. Klein R. M. (2011). Are there bilingual advantages on nonlinguistic interference tasks? Implications for the plasticity of executive control processes. Psychon. Bull. Rev. 18, 625–658. doi: 10.3758/s13423-011-0116-7, PMID: 21674283

[ref57] Hirosh Z. Degani T. (2018). Direct and indirect effects of multilingualism on novel language learning: An integrative review. Psychon. Bull. Rev. 25, 892–916. doi: 10.3758/s13423-017-1315-7, PMID: 28547538

[ref58] Huang Y. Su L. Ma Q. (2020). The Stroop effect: An activation likelihood estimation meta-analysis in healthy young adults. Neurosci. Lett. 716:134683. doi: 10.1016/j.neulet.2019.134683, PMID: 31830505

[ref59] Ito T. Kulkarni K. R. Schultz D. H. Mill R. D. Chen R. H. Solomyak L. I.. (2017). Cognitive task information is transferred between brain regions via resting-state network topology. Nat. Commun. 8:1027. doi: 10.1038/s41467-017-01000-w, PMID: 29044112 PMC5715061

[ref60] Kanasi E. Ayilavarapu S. Jones J. (2016). The aging population: Demographics and the biology of aging. Periodontol. 72, 13–18. doi: 10.1111/prd.12126, PMID: 27501488

[ref61] Kane M. J. Engle R. W. (2003). Working-memory capacity and the control of attention: The contributions of goal neglect, response competition, and task set to Stroop interference. J. Exp. Psychol. Gen. 132, 47–70. doi: 10.1037/0096-3445.132.1.47, PMID: 12656297

[ref62] Kaushanskaya M. Blumenfeld H. K. Marian V. (2020). The Language Experience and Proficiency Questionnaire (LEAP-Q): Ten years later. Biling. Lang. Congn. 23, 945–950. doi: 10.1017/S1366728919000038, PMID: 33628083 PMC7899192

[ref63] Klimova B. Valis M. Kuca K. (2017). Cognitive decline in normal aging and its prevention: A review on non-pharmacological lifestyle strategies. Clin. Interv. Aging 12, 903–910. doi: 10.2147/CIA.S132963 28579767 PMC5448694

[ref64] Korenar M. Pliatsikas C. (2023). “Second language acquisition and neuroplasticity: Insights from the Dynamic Restructuring Model” in The Routledge Handbook of Second Language Acquisition and Neurolinguistics (New York: Routledge), 191–203.

[ref65] Kroll J. F. Bialystok E. (2013). Understanding the consequences of bilingualism for language processing and cognition. J. Cogn. Psychol. 25, 497–514. doi: 10.1080/20445911.2013.799170, PMID: 24223260 PMC3820916

[ref66] Kroll J. F. Bobb S. C. Misra M. Guo T. (2008). Language selection in bilingual speech: Evidence for inhibitory processes. Acta Psychol. 128, 416–430. doi: 10.1016/j.actpsy.2008.02.001, PMID: 18358449 PMC2585366

[ref67] Li P. Legault J. Litcofsky K. A. (2014). Neuroplasticity as a function of second language learning: Anatomical changes in the human brain. Cortex 58, 301–324. doi: 10.1016/j.cortex.2014.05.001, PMID: 24996640

[ref68] Li X. Morgan P. S. Ashburner J. Smith J. Rorden C. (2016). The first step for neuroimaging data analysis: DICOM to NIfTI conversion. J. Neurosci. Methods 264, 47–56. doi: 10.1016/j.jneumeth.2016.03.001, PMID: 26945974

[ref69] Li P. Xu Q. (2023). Computational modeling of bilingual language learning: Current models and future directions. Lang. Learn. 73, 17–64. doi: 10.1111/lang.12529, PMID: 38385119 PMC10881204

[ref70] Liberati G. Raffone A. Olivetti Belardinelli M. (2012). Cognitive reserve and its implications for rehabilitation and Alzheimer’s disease. Cogn. Process. 13, 1–12. doi: 10.1007/s10339-011-0410-3, PMID: 21643921

[ref71] Lin Y. T. Lai Y. H. (2024). Stroop color-word test performance of Chinese-speaking persons with Alzheimer's dementia. Int. J. Gerontol. 18, 80–84. doi: 10.6890/IJGE.202404_18(2).0004

[ref72] Lopez O. L. Kuller L. H. (2019). Epidemiology of aging and associated cognitive disorders: Prevalence and incidence of Alzheimer’s disease and other dementias. *Handb. Clin. Neurol*. 167, 139–148. doi: 10.1016/B978-0-12-804766-8.00009-1 31753130

[ref73] Love J. Selker R. Marsman M. Jamil T. Dropmann D. Verhagen J.. (2019). JASP: graphical statistical software for common statistical designs. J. Stat. Softw. 88, 1–17. doi: 10.18637/jss.v088.i02, PMID: 33634423

[ref74] MacLeod C. M. (1991). Half a century of research on the Stroop effect: an integrative review. Psychol. Bull. 109, 163–203. doi: 10.1037/0033-2909.109.2.163, PMID: 2034749

[ref75] Manelis A. Reder L. M. (2014). Effective connectivity among the working memory regions during preparation for and during performance of the n-back task. Front. Hum. Neurosci. 8:593. doi: 10.3389/fnhum.2014.00593, PMID: 25140143 PMC4122182

[ref76] Marian V. Blumenfeld H. K. Kaushanskaya M. (2007). The Language Experience and Proficiency Questionnaire (LEAP-Q): Assessing Language Profiles in Bilinguals and Multilinguals. J. Speech Lang. Hear. Res. 50, 940–967. doi: 10.1044/1092-4388(2007/067), PMID: 17675598

[ref77] Meltzer J. A. Kates Rose M. Le A. Y. Spencer K. A. Goldstein L. Gubanova A.. (2023). Improvement in executive function for older adults through smartphone apps: A randomized clinical trial comparing language learning and brain training. Aging Neuropsychol. Cognit. 30, 150–171. doi: 10.1080/13825585.2021.1991262, PMID: 34694201

[ref78] Menon V. D’Esposito M. (2022). The role of PFC networks in cognitive control and executive function. Neuropsychopharmacology 47, 90–103. doi: 10.1038/s41386-021-01152-w, PMID: 34408276 PMC8616903

[ref79] Mill R. D. Gordon B. A. Balota D. A. Cole M. W. (2020). Predicting dysfunctional age-related task activations from resting-state network alterations. NeuroImage 221:117167. doi: 10.1016/j.neuroimage.2020.117167, PMID: 32682094 PMC7810059

[ref80] Momková E. Jurásová K. I. (2017). Performance of bilingual individuals in psychodiagnostic testing of cognitive abilities using their first and second languages. Psychol. Contexts 8, 66–85.

[ref81] Morbelli S. Arnaldi D. Capitanio S. Picco A. Buschiazzo A. Nobili F. (2013). Resting metabolic connectivity in Alzheimer’s disease. Clin. Translat. Imaging 1, 271–278. doi: 10.1007/s40336-013-0027-x, PMID: 38999798

[ref82] Nelson M. E. Jester D. J. Petkus A. J. Andel R. (2021). Cognitive reserve, Alzheimer’s neuropathology, and risk of dementia: a systematic review and meta-analysis. Neuropsychol. Rev. 31, 233–250. doi: 10.1007/s11065-021-09478-4, PMID: 33415533 PMC7790730

[ref83] Niendam T. A. Laird A. R. Ray K. L. Dean Y. M. Glahn D. C. Carter C. S. (2012). Meta-analytic evidence for a superordinate cognitive control network subserving diverse executive functions. Cogn. Affect. Behav. Neurosci. 12, 241–268. doi: 10.3758/s13415-011-0083-5, PMID: 22282036 PMC3660731

[ref84] Nilsson J. Berggren R. Garzón B. Lebedev A. V. Lövdén M. (2021). Second language learning in older adults: effects on brain structure and predictors of learning success. Front. Aging Neurosci. 13:666851. doi: 10.3389/fnagi.2021.666851 34149398 PMC8209301

[ref85] Olson D. J. (2024). A systematic review of proficiency assessment methods in bilingualism research. Int. J. Biling. 28, 163–187. doi: 10.1177/13670069231153720, PMID: 33556665

[ref86] Oosterhuis E. J. Slade K. Smith E. May P. J. Nuttall H. E. (2023). Getting the brain into gear: an online study investigating cognitive reserve and word-finding abilities in healthy ageing. PLoS One 18:e0280566. doi: 10.1371/journal.pone.0280566, PMID: 37079604 PMC10118119

[ref87] Osareme J. Muonde M. Maduka C. P. Olorunsogo T. O. Omotayo O. (2024). Demographic shifts and healthcare: A review of aging populations and systemic challenges. Int. J. Sci. Res. Archive 11, 383–395. doi: 10.30574/ijsra.2024.11.1.0067

[ref88] Power J. D. Barnes K. A. Snyder A. Z. Schlaggar B. L. Petersen S. E. (2012). Spurious but systematic correlations in functional connectivity MRI networks arise from subject motion. NeuroImage 59, 2142–2154. doi: 10.1016/j.neuroimage.2011.10.018, PMID: 22019881 PMC3254728

[ref89] Rao A. Chatterjee P. Rao A. R. Dey A. B. (2023). A cross-sectional study of various memory domains in normal ageing population and subjective cognitive decline using PGIMS and Stroop color-word test. J. Indian Acad. Geriatrics 19.

[ref90] Rodríguez-Pujadas A. Sanjuán A. Fuentes P. Ventura-Campos N. Barrós-Loscertales A. Ávila C. (2014). Differential neural control in early bilinguals and monolinguals during response inhibition. Brain Lang. 132, 43–51. doi: 10.1016/j.bandl.2014.03.003, PMID: 24735970

[ref91] Savarimuthu A. Ponniah R. J. (2024). Cognition and cognitive reserve. Integr. Psychol. Behav. Sci. 58, 483–501. doi: 10.1007/s12124-024-09821-3 38279076

[ref92] Scarmeas N. Stern Y. (2003). Cognitive reserve and lifestyle. J. Clin. Exp. Neuropsychol. 25, 625–633. doi: 10.1076/jcen.25.5.625.14576, PMID: 12815500 PMC3024591

[ref93] Scarpina F. Tagini S. (2017). The Stroop Color and Word Test. Front. Psychol. 8:557. doi: 10.3389/fpsyg.2017.00557, PMID: 28446889 PMC5388755

[ref95] Schultz D. H. Ito T. Cole M. W. (2022). Global connectivity fingerprints predict the domain generality of multiple-demand regions. Cereb. Cortex. 32, 4464–4479. doi: 10.1093/cercor/bhab495 35076709 PMC9574240

[ref96] Schweizer T. A. Ware J. Fischer C. E. Craik F. I. M. Bialystok E. (2012). Bilingualism as a contributor to cognitive reserve: evidence from brain atrophy in Alzheimer’s disease. Cortex 48, 991–996. doi: 10.1016/j.cortex.2011.04.009, PMID: 21596373

[ref97] Simonet M. (2014). Long-term training-induced behavioural and structural plasticity of inhibitory control in elite fencers. Available at: https://www.semanticscholar.org/paper/Long-term-training-induced-behavioural-and-of-in-Simonet/6a7279480725662209896894432ee2dc9ab488ad

[ref98] Šneidere K. Zdanovskis N. Mondini S. Stepens A. (2024). Relationship between lifestyle proxies of cognitive reserve and cortical regions in older adults. Front. Psychol. 14:1308434. doi: 10.3389/fpsyg.2023.1308434 38250107 PMC10797127

[ref99] Song Y. Hakoda Y. (2015). An fMRI study of the functional mechanisms of Stroop/reverse-Stroop effects. Behav. Brain Res. 290, 187–196. doi: 10.1016/j.bbr.2015.04.047, PMID: 25952963

[ref100] Stålhammar J. Hellström P. Eckerström C. Wallin A. (2022). Neuropsychological test performance among native and non-native Swedes: Second language effects. Arch. Clin. Neuropsychol. 37, 826–838. doi: 10.1093/arclin/acaa043, PMID: 32722802 PMC9113439

[ref101] Stern Y. (2002). What is cognitive reserve? Theory and research application of the reserve concept. J. Int. Neuropsychol. Soc. 8, 448–460. doi: 10.1017/S1355617702813248, PMID: 11939702

[ref102] Stern Y. (2009). Cognitive reserve☆. Neuropsychologia 47, 2015–2028. doi: 10.1016/j.neuropsychologia.2009.03.004, PMID: 19467352 PMC2739591

[ref103] Stern Y. (2021). How can cognitive reserve promote cognitive and neurobehavioral health? Archives Clin. Neuropsychol. 36, 1291–1295. doi: 10.1093/arclin/acab049, PMID: 34651645 PMC8517622

[ref104] Stern Y. Arenaza-Urquijo E. M. Bartrés-Faz D. Belleville S. Cantilon M. Chetelat G.. (2020). Whitepaper: Defining and investigating cognitive reserve, brain reserve, and brain maintenance. Alzheimers Dement. 16, 1305–1311. doi: 10.1016/j.jalz.2018.07.219, PMID: 30222945 PMC6417987

[ref105] Stroop J. R. (1935). Studies of interference in serial verbal reactions. J. Exp. Psychol. 18, 643–662. doi: 10.1037/h0054651, PMID: 33211511

[ref106] Surrain S. Luk G. (2019). Describing bilinguals: A systematic review of labels and descriptions used in the literature between 2005–2015. Biling. Lang. Congn. 22, 401–415. doi: 10.1017/S1366728917000682

[ref107] Teubner-Rhodes S. Bolger D. J. Novick J. M. (2019). Conflict monitoring and detection in the bilingual brain. Biling. Lang. Congn. 22, 228–252. doi: 10.1017/S1366728917000670, PMID: 33718987

[ref108] Tucker M. Stern Y. (2011). Cognitive Reserve in Aging. Curr. Alzheimer Res. 8, 354–360. doi: 10.2174/156720511795745320, PMID: 21222591 PMC3135666

[ref109] Waldie K. E. Badzakova-Trajkov G. Miliivojevic B. Kirk I. J. (2009). Neural activity during Stroop colour-word task performance in late proficient bilinguals: a functional magnetic resonance imaging study. Psychol. Neurosci. 2, 125–136. doi: 10.3922/j.psns.2009.2.004

[ref110] Wong P. C. M. Ou J. Pang C. W. Y. Zhang L. Tse C. S. Lam L. C. W.. (2019). Language Training Leads to Global Cognitive Improvement in Older Adults: A Preliminary Study. J. Speech Lang. Hear. Res. 62, 2411–2424. doi: 10.1044/2019_JSLHR-L-18-0321, PMID: 31251679

[ref111] Work N. (2014). Rosetta Stone: Compatible with Proficiency Oriented Language Instruction and Blended Learning. J. Technol. Teach. Learn. 10, 35–52.

[ref112] Ye Z. Zhou X. (2009). Executive control in language processing. Neurosci. Biobehav. Rev. 33, 1168–1177. doi: 10.1016/j.neubiorev.2009.03.003, PMID: 19747595

[ref113] Zheng Y. (2024). “The Role of Technology in English Language Education: Online Platforms, Apps, and Virtual Reality” in Proceedings of the 3rd International Conference on Education, Language and Art (ICELA 2023) (Vol. 831, pp. 255–261). eds. Zhu S. Baldini A. L. Hong Y. Xu Z. Syed Mohammed S. F. (Atlantis Press SARL). Available at: https://www.atlantis-press.com/proceedings/icela-23/publishing

